# *Lactobacillus plantarum* Dynamically Changes the Fermentation Characteristics and Active Ingredients of Summer/Autumn Tea Leaves

**DOI:** 10.1002/fsn3.71773

**Published:** 2026-04-17

**Authors:** Yimei Luo, Yuangan Qian, Yangwen Ou, Shimin Tian, Yixiao Xie, Yulong Zheng, Jun Hao, Hong Sun, Fuyu Yang

**Affiliations:** ^1^ College of Animal Science Guizhou University Guiyang China; ^2^ College of Grassland Science and Technology China Agricultural University Beijing China

**Keywords:** active ingredients, anaerobic tea fermentation, free amino acids, *Lactobacillus plantarum*, microbial community

## Abstract

Yields of summer/autumn tea in China are relatively high, but its utilization and market value remain limited because of the pronounced bitterness and inferior flavor. In southwestern China, fresh summer/autumn tea leaves are traditionally pickled through anaerobic fermentation. In this study, fresh summer/autumn tea leaves were subjected to controlled anaerobic fermentation with *Lactiplantibacillus plantarum* (LP) inoculation, and the dynamic changes in nutritional quality, fermentation characteristics, active ingredients, and microbial community were evaluated. Compared with the noninoculated treatment (CK), LP treatment significantly reduced levels of water‐soluble carbohydrates (WSCs), acetic acid (AA), and propionic acid (PA), while increasing the acid detergent fiber (ADF) and ammonia nitrogen (NH_3_–N) contents in fermented tea leaves (all *p* < 0.05). The total free amino acids (FAA) content peaked on Day 30, reaching 8.15% and 7.94% for the CK and LP treatments, respectively. In addition, the contents of the flavor‐related FAA, including Glu and Ala, in the LP treatment were increased by 241.74% and 268.26% on Day 15, respectively. LP inoculation markedly elevated the relative abundance of *Lactiplantibacillus* and reduced the relative abundance of *Listeria*. Overall, LP inoculation improved the fermentation quality, enhanced the levels of bioactive and nutritional components, enriched specific flavor‐related FAA, and altered the microbial community by promoting the LAB abundance. These findings provide a theoretical basis for the efficient utilization and value promotion of summer/autumn tea leaves in southwest China.

## Graphical abstract

*Lactiplantibacillus plantarum* modulates summer/autumn tea fermentation, reshaping microbial communities to enrich umami and sweet amino acids while suppressing harmful bacteria, thereby effectively improving flavor quality.

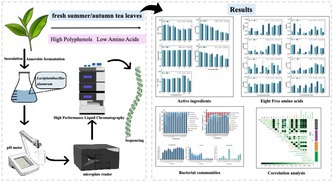

## 1 Introduction

Tea is the second most commonly consumed beverage globally, surpassed only by water. In 2023, China's total tea output exceeded 3.5 million metric tons from more than 3.4 million hectares of plantations (National Bureau of Statistics of China 2023). Tea can be divided into spring, summer, and autumn tea; summer/autumn tea accounts for more than 60% of the annual production (Xu et al. 2022). However, compared to spring tea, summer/autumn tea is of inferior quality because it contains higher levels of polyphenols and lower levels of amino acids than spring tea (Liu et al. 2023). These compositional characteristics are associated with a bitter/astringent flavor and reduced freshness, thereby compromising overall quality. These characteristics and the resulting sensory drawbacks severely restrict the processing utilization rate and market value of summer/autumn tea. Therefore, the development of appropriate processing technology to reduce the excessive bitterness of summer/autumn tea, and enhancing its nutritional quality and bioactive components, is a key issue that remains to be addressed in the tea industry.

Fermentation has been widely recognized as a key biotechnological approach for enhancing the sensory properties and biological activity of tea materials. In recent years, tea‐based fermented beverages and ingredients have attracted increasing attention (Fang et al. 2026; Cheng et al. 2025). During fermentation, microbial metabolism and enzymatic action reduce the levels of catechins, phenolic acids, and caffeine, thereby alleviating the bitterness and astringency of the tea infusion and improving the overall taste (Ye et al. 2022). Meanwhile, microbial transformation reshapes the aroma composition of tea leaves by promoting the formation and release of terpenic alcohols and ketones, shifting the aroma from predominantly green notes to rich floral, fruity, and sweet notes (Zhang, Huang, et al. 2024).

Pickled tea, a traditional fermented tea product from ethnic minority regions in southwestern China, is typically made from fresh tea leaves through anaerobic fermentation, with its quality formation primarily driven by epiphytic microorganisms on the leaf surface. Among these, lactic acid bacteria (LAB) are recognized as the core functional microbiota and are highly valued for their metabolic diversity and probiotic potential (Wu et al. 2025; Guan et al. 2024). In plant fermentation matrices, LAB utilize carbohydrates to produce organic acids, which not only extends the shelf life and improves the flavor of fermented products but also promotes the production and accumulation of various health‐related bioactive substances. In tea leaf fermentation, LAB secrete various enzymes that promote the release of phenolic compounds, amino acids, and dietary fiber‐related micronutrients (Cheng et al. 2025). LAB also drive biotransformation reactions such as deglycosylation, deesterification, and demethylation, which can convert tea‐derived phenolic acids, flavonoids, catechins, theaflavins, and thearubigins into derivatives with higher bioavailability and bioactivity (Fang et al. 2026). These transformations are often accompanied by decreased contents of caffeine and certain catechins and increased levels of flavor‐related free amino acids (FAA) and gallic acid (GA), thereby improving the smoothness and mellow taste of tea infusion and enhancing sweetness (Horie and Iwahashi 2023; Wen et al. 2023; Zhou et al. 2025). Moreover, LAB synthesize functional metabolites, including short‐chain fatty acids, exopolysaccharides, and bioactive peptides (Zang et al. 2025). Thus, LAB enhance the sensory characteristics and nutritional value of tea through the synergistic pathways of biosynthesis and biological release (Cheng et al. 2025).

However, traditional pickled tea production mainly relies on spontaneous fermentation by epiphytic microorganisms, which is susceptible to contamination by undesirable microorganisms, resulting in poor process controllability and unstable product quality. In addition, although the high levels of tannins and polyphenols in tea leaves exhibit strong antioxidant activity, they inhibit the growth and metabolic activity of LAB. It has recently been shown that the tea matrix reduces the abundance of *Lactobacillus plantarum* (LP) during fermentation, thereby influencing the biotransformation of flavor precursors, including amino acids and catechins (Lei et al. 2024; Zhou et al. 2024). Currently, research on the directional optimization of summer/autumn tea fermentation through artificial inoculation of LP remains inadequate, and the core mechanism by which LP regulates the sensory and nutritional quality of summer/autumn tea has not been systematically clarified.

Therefore, in this study, fresh summer/autumn tea leaves were used as the fermentation substrate and inoculated with exogenous LP for controlled anaerobic fermentation. The dynamic changes in fermentation characteristics, nutritional quality, bioactive component composition, and microbial community structure were systematically investigated. This study aimed to clarify how LP regulates the contents of total FAA and flavor‐related FAA and key polyphenols and how these changes affect the flavor formation in fermented tea.

## 2 Methods

### 2.1 Experimental Design and Treatments

The summer/autumn tea leaves were collected on June 27, 2022, from Guizhou Classic Yunwu Tea Industry Co. Ltd. (Guizhou, China). The fresh leaves were withered under ventilated conditions until the moisture content reached approximately 75% and then fully rolled using a kneading machine. LP (viable count > 3.5 × 10^8^ CFU/g), characterized by rapid acid production, was purchased from Beijing Biobw Biotechnology Co. Ltd. (Beijing, China) as a fermentation inoculation.

The experiment followed a completely randomized design with a 2 × 6 factorial design, consisting of two inoculation treatments and six fermentation days (1, 3, 7, 15, 30, and 45 day). The inoculation treatments are as follows: (1) LP treatment, in which the inoculum is applied at 1 × 10^6^ CFU/g fresh matter (FM), with LP powder pre‐dissolved in 10 mL of sterile water before application. (2) Control treatment (CK), in which an equal volume of sterile water was added. For each treatment, 300 g of prepared tea leaves were thoroughly mixed with the corresponding inoculum, packed into polyethylene vacuum bags (18 cm × 25 cm, 0.08 mm thickness), and sealed using a vacuum sealer (Shineye PW300) to maintain anaerobic conditions. In total, 60 bags were prepared (2 additive treatments × 6 periods × 5 replicates) and stored in a constant temperature room at 25°C ± 1°C. On Days 1, 3, 7, 15, 30, and 45, five bags of each treatment were randomly selected for subsequent analysis.

### 2.2 Fermentation Characteristics and Chemical Composition

After anaerobic storage, the samples were dried at 60°C for 48 h to constant weight, and the dry matter (DM) content was measured. The dried samples were crushed and stored in self‐sealing bags for the determination of other nutritional indicators. Specifically, the crude protein (CP) content was determined by the Kjeldahl method (AOAC International 2012). The contents of neutral detergent fiber (NDF) and acid detergent fiber (ADF) were determined by the Van Soest detergent fiber method (Van Soest et al. 1991). The water‐soluble carbohydrates (WSC) content was measured according to the method of Anthrone colorimetry (Kha and Chaudhry 2010). Briefly, 20 g of sample was thoroughly mixed with 180 mL of sterile distilled water, and the filtrate was obtained after filtration through four layers of nylon gauze. Then 40 mL of the filtrate was mixed with 10 mL of 25% trichloroacetic acid and passed overnight at 4°C. After centrifugation at 18,000*g* at 4°C for 15 min, the supernatant was collected to determine the nonprotein nitrogen (NPN) content (AOAC International 2012). The contents of GA, catechins, and caffeine were determined by high‐performance liquid chromatography (Xu et al. 2020).

To analyze the fermentation characteristics of anaerobic preservation of tea leaves, a total of 10 g of anaerobic fermentation sample was mixed with 90 mL of sterilized water, incubated at 4°C overnight, and then filtered through four layers of cheesecloth. The pH was determined using a pH meter (Rex PHS‐3E, INESA Scientific Instrument Co. Ltd., Shanghai, China). The content of ammonia nitrogen (NH_3_–N) was determined by the phenol‐sodium hypochlorite colorimetric method, and the contents of organic acids, including lactic acid (LA), acetic acid (AA), propionic acid (PA), and butyric acid (BA), were determined by high‐performance liquid chromatography (Lin et al. 2021).

### 2.3 Total Tea Polyphenols (TPs) Content

Preparation of the test solution: One gram of tea powder (crushed through a 60‐mesh sieve) was weighed and placed in a 250 mL conical flask. 90 mL of boiling water was added, and the flask was placed in a boiling water bath and oscillated every 10 min for a total of 45 min. After extraction was completed, the mixture was filtered while hot, and the tea infusion was diluted to 100 mL once cooled to room temperature at 25°C ± 1°C. A 1 mL aliquot of the test solution was transferred into a volumetric tube, mixed with 5 mL of Folin–Ciocalteu reagent, and shaken thoroughly. After a precise reaction time of 5 min, 4 mL of 7.5% Na_2_CO_3_ solution was added, and the mixture was shaken thoroughly again. The solution was then incubated at room temperature (25°C ± 1°C) for 60 min. The absorbance was measured at 760 nm using a Varioskan LUX microplate reader (Thermo Fisher Scientific, Waltham, MA, USA). A standard calibration curve was established using GA solutions of different concentrations to calculate the total TPs content in tea samples (Xu et al. 2020).

### 2.4 Analysis of Flavor‐Related FAA

Eight flavor‐related FAA were quantified: alanine (Ala), aspartic acid (Asp), γ‐aminobutyric acid (GABA), glutamic acid (Glu), glycine (Gly), lysine (Lys), theanine (Thea), and tryptophan (Trp). Preparation of flavor‐related FAA samples: A 2 g sample was placed in a 250 mL conical flask, and 200 mL of boiling water was added. The mixture was heated in a boiling water bath for 10 min and then cooled to room temperature at 25°C ± 1°C. A 2 mL aliquot of the supernatant was filtered through a 0.22 μm syringe filter membrane. The concentrations of the eight flavor‐related FAA in the samples were measured using an automatic amino acid analyzer (Sykam S‐433, Eresing, Germany) (Chen et al. 2020).

### 2.5 Bacterial Community Analysis

The sequencing and bioinformatics analysis of microorganisms in tea leaves after anaerobic fermentation were performed by Beijing Novogene Technology Co. Ltd. (Beijing, China). The PCR amplification and subsequent analyses were conducted using DNA extracted from all samples. Universal bacterial 16S rRNA (V3–V4 region) primers were used for PCR amplification, with the specific sequences: 5′‐ACTCCTACGGGAGGCAGCA‐3′ (forward) and 5′‐GGACTACHVGGGTWTCTAAT‐3′ (reverse). A paired‐end sequencing library was then constructed using high‐throughput sequencing technology. Microbial community sequencing was performed on the Illumina HiSeq platform with paired‐end sequencing (PE150). A short‐insert library was constructed for sequencing, and the sequencing results included alpha diversity indices (Ace, Chao1, Shannon, and Simpson indices) and microbial community structure analysis (He et al. 2020).

### 2.6 Statistical Analysis

Two‐way analysis of variance (ANOVA) was performed using SPSS 26.0 software (SPSS Inc., Chicago, IL, USA) to evaluate the effects of inoculation treatments, fermentation days, and their interaction. Differences among treatment means were compared using Duncan's multiple range test (*p* < 0.05). Pearson correlation analysis was performed to assess the relationships among bacterial genera, nutritional components, fermentation characteristics, and active ingredients. The results were calculated and visualized using R software (version 4.5.2).

## 3 Results

### 3.1 The Raw Material

Table 1 shows that the pH of fresh summer/autumn tea leaves was 4.80 and the DM content was 29.81% FM. The CP and WSC levels were 35.48% and 2.65%, respectively. Other components included 9.70% NPN, 23.00% NDF, 17.65% ADF, and 1.00% NH_3_–N.

**TABLE 1:** Chemical composition of the tea leaves raw material.

Items	Content
pH (%DM)	4.80 ± 0.03
DM (% FM)	29.81 ± 0.20
CP (%DM)	35.48 ± 0.05
NPN (%TN)	9.70 ± 0.08
WSC (%DM)	2.65 ± 0.11
NDF (%DM)	22.98 ± 1.00
ADF (%DM)	17.65 ± 0.76
NH_3_–N (%DM)	0.98 ± 0.08

*Note:* Data are mean ± standard deviation of triplicate determinations.

Abbreviations: ADF, acid detergent fiber; CP, crude protein; DM, dry matter; FM, fresh matter; NDF, neutral detergent fiber; NH_3_–N, ammoniacal nitrogen; NPN, nonprotein nitrogen; TN, total nitrogen; WSC, water‐soluble carbohydrate.

The active ingredients of fresh tea leaves are listed in Table 2. The TPs, GA, caffeine, catechin, and total FAA contents were 35.54%, 0.15%, 3.51%, 0.50%, and 4.40%, respectively. The levels of eight common amino acids (Ala, Asp, GABA, Glu, Gly, Lys, Thea, and Trp) were 0.02%, 0.27%, 0.03%, 0.17%, 0.34%, 0.05%, 1.03%, and 0.06%, respectively.

**TABLE 2:** Active ingredients composition of the tea leaves raw material (%DM).

Items	Content
Tea polyphenols	35.54 ± 1.22
Gallic acid	0.15 ± 0.01
Caffeine	3.51 ± 0.02
Catechins	0.50 ± 0.02
Free amino acids	4.40 ± 0.66
Alanine	0.02 ± 0.00
Aspartic acid	0.27 ± 0.04
γ‐Aminobutyric acid	0.03 ± 0.00
glutamic acid	0.17 ± 0.09
Glycine	0.34 ± 0.04
Lysine	0.05 ± 0.00
Theanine	1.03 ± 0.34
Tryptophan	0.06 ± 0.01

### 3.2 Chemical Composition of Tea Leaves

Table 3 lists the chemical compositions of the tea leaves with or without LP inoculation during anaerobic fermentation. Such addition significantly affected the DM and WSC contents (*p* < 0.05) as fermentation proceeded, and also the levels of CP, NDF, ADF, and WSC (*p* < 0.05). Significant interactions between the treatment and fermentation days were observed for DM and CP (*p* < 0.05). Compared to the CK, the average DM and WSC contents decreased by 0.75% and 7.02%, respectively, but the average concentrations of CP, NPN, and NDF were not affected (*p* > 0.05). As fermentation time proceeded, the DM and WSC contents tended to decrease, and those of CP, NPN, ADF, and NDF to increase in both the CK and the LP treatment.

**TABLE 3:** The chemical composition of tea leaves with or without *Lactobacillus plantarum* inoculation during anaerobic fermentation.

Items		DM	CP	NPN	NDF	ADF	WSC
		%FM	%DM	%TN	%DM	%DM	%DM
CK	1 days	30.13a	34.45bc	9.42	21.74	16.96	2.65
	3 days	29.49bc	34.17c	8.74	23.38	17.59	2.47
	7 days	29.72b	34.47bc	9.50	21.37	15.89	2.44
	15 days	29.00d	34.37bc	9.52	22.31	13.68	2.42
	30 days	29.25 cd	34.69b	10.39	21.77	14.06	2.48
	45 days	28.16e	35.15a	10.59	26.00	17.66	2.07
LP	1 days	30.13a	34.55bc	9.35	21.67	16.26	2.22
	3 days	29.63bc	34.59bc	9.47	23.20	18.69	2.23
	7 days	29.66b	34.39bc	9.34	23.98	18.13	2.50
	15 days	29.50bc	34.16c	9.24	23.33	14.68	2.39
	30 days	26.98f	35.10a	9.26	23.41	14.62	2.37
	45 days	28.52e	34.65b	9.75	27.51	19.53	1.79
SEM		0.036	0.038	0.111	0.331	0.277	0.029
Treatment (*T*)							
CK		29.29a	34.55	9.69	22.76	15.97	2.42a
LP		29.07b	34.58	9.40	23.85	16.99	2.25b
Fermentation days (*F*)							
1 days		30.13a	34.50b	9.39ab	21.71b	16.61a	2.44a
3 days		29.56b	34.38b	9.11b	23.29b	18.14a	2.35a
7 days		29.69b	34.43b	9.42ab	22.67b	17.01a	2.41a
15 days		29.25c	34.27b	9.38ab	22.82b	14.18b	2.42a
30 days		28.11d	34.89a	9.82ab	22.59b	14.34b	2.37a
45 days		28.34d	34.90a	10.17a	26.76a	18.60a	1.93b
Significance (*p*)							
*T*		**	NS	NS	NS	NS	**
*F*		***	***	NS	**	***	***
*T* × *F*		***	**	NS	NS	NS	NS

*Note:* ^a–e^ means differences of the same column (*p* < 0.05).

Abbreviations: ADF, acid detergent fiber; CK, control; CP, crude protein; DM, dry matter; *F*, fermentation days; FM, fresh matter; LP, *L. plantarum* ; NDF, neutral detergent fiber; NPN, nonprotein nitrogen; NS, not significant; SEM, standard error of the mean; *T* × *F*, interaction between treatment and fermentation days; *T*, treatments; TN, total nitrogen; WSC, water‐soluble carbohydrate.

The significance level is shown as follows: *p < 0.05; **p < 0.01; ***p < 0.001.

### 3.3 Fermentation Quality

The fermentation qualities of the tea leaves with or without LP inoculation during anaerobic fermentation are shown in Table 4. For both the CK and other treatments, the levels of AA, PA, and NH_3_–N changed markedly (*p* < 0.05). The fermentation time and additive treatment significantly affected the AA, LA/AA, PA, and NH_3_–N levels (*p* < 0.05). Compared to the CK, the average LA, AA, and PA contents during LP treatment decreased by 22.58%, 36.81%, and 16.87%, respectively, but the NH_3_‐N content increased by 5.43%. As fermentation proceeded, the pH and the LA/AA decreased on LP treatment. Compared to Day 1, the decreases were 3.53% and 44.62% by Day 45, respectively. In contrast, the LA, AA, and NH_3_–N contents during LP treatment increased by 103.17%, 267.35%, and 48.54% on Day 45, respectively, compared to Day 1. BA was never detected in all treatments.

**TABLE 4:** The fermentation quality of tea leaves with or without *Lactobacillus plantarum* inoculation during anaerobic fermentation.

Items		pH	LA	AA	LA/AA	PA	BA	NH_3_–N
			%DM	%DM	%DM	%DM	%DM	%DM
CK	1 days	4.36e	0.87	0.38c	2.33a	0.02f	ND	5.06g
	3 days	4.58a	0.56	1.72b	0.54de	0.08f	ND	5.79fg
	7 days	4.47bcd	1.31	4.27a	0.31e	0.35d	ND	6.52ef
	15 days	4.48bc	2.23	4.34a	0.51de	0.73c	ND	7.11de
	30 days	4.41cde	1.27	4.11a	0.31e	1.53b	ND	8.37b
	45 days	4.48bc	1.20	3.59a	0.33e	2.30a	ND	8.07bc
LP	1 days	4.53ab	0.63	0.98bc	0.65de	ND	ND	5.15 g
	3 days	4.53ab	0.81	1.11bc	0.86 cd	ND	ND	5.91 fg
	7 days	4.39de	0.94	0.65c	1.46b	0.25e	ND	9.10a
	15 days	4.48bc	1.04	0.92bc	1.13bc	0.83c	ND	7.27cde
	30 days	4.47bcd	1.03	4.36a	0.24e	0.82c	ND	8.09bc
	45 days	4.37e	1.28	3.60a	0.36e	2.25a	ND	7.65bcd
SEM		0.007	0.088	0.082	0.040	0.009	ND	0.086
Treatment (*T*)								
CK		4.46	1.24	3.07a	0.72	0.83a	ND	6.82b
LP		4.47	0.96	1.94b	0.78	0.69b	ND	7.19a
Fermentation days (*F*)								
1 days		4.45b	0.75	0.68e	1.49a	0.01e	ND	5.10d
3 days		4.56a	0.69	1.42d	0.70b	0.04e	ND	5.85c
7 days		4.43b	1.12	2.46c	0.88b	0.30d	ND	7.81a
15 days		4.48b	1.63	2.63c	0.82b	0.78c	ND	7.19b
30 days		4.44b	1.15	4.24a	0.27c	1.17b	ND	8.23a
45 days		4.43b	1.24	3.60b	0.35c	2.28a	ND	7.86a
Significance (*p*)								
*T*		NS	NS	***	NS	***	NS	*
*F*		***	NS	***	***	***	NS	***
*T* × *F*		***	NS	***	***	***	NS	***

*Note:* ^a–e^ means differences of the same column (*p* < 0.05). The significance level is shown as follows *p < 0.05; **p < 0.01; ***p < 0.001.

Abbreviations: AA, acetic acid; BA, butyric acid; CK, control; DM, dry matter; *F*, fermentation days; LA, lactic acid; LP, *L. plantarum* ; ND, not detected; NH_3_–N, ammonia nitrogen; NS, not significant; PA, propionic acid; SEM, standard error of the mean; *T* × *F*, interaction between treatment and fermentation days; *T*, treatments; TN, total nitrogen.

### 3.4 Active Ingredients

**FIGURE 1:**
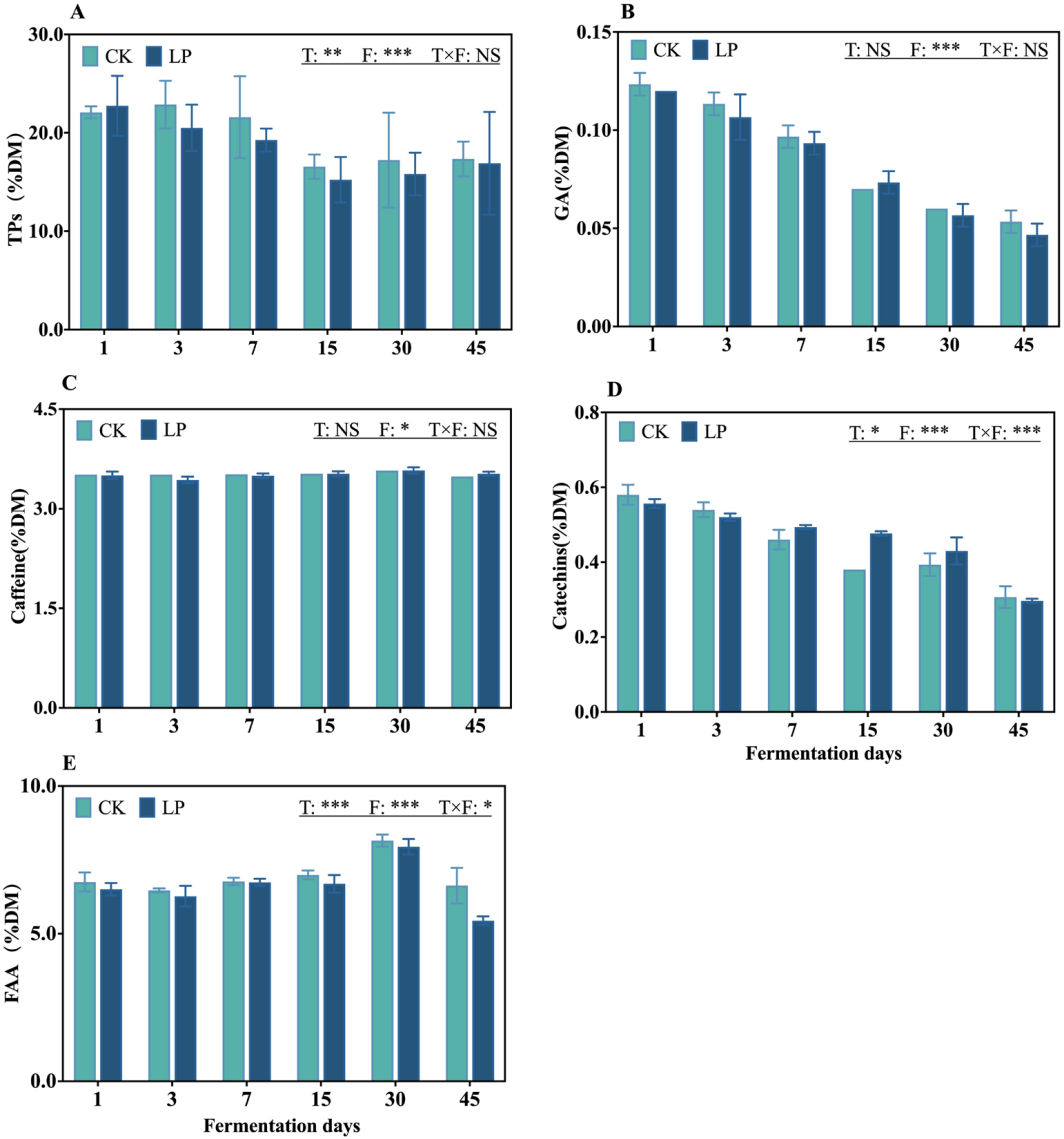
The active ingredients of tea leaves with or without *Lactobacillus plantarum* inoculation during anaerobic fermentation. (A) Tea polyphenols (TPs); (B) Gallic acid (GA); (C) Caffeine; (D) Catechins; (E) Free amino acids. CK, control; *F*, fermentation days; LP, *L. plantarum* ; *T* × *F*, interaction between treatment and fermentation days; *T*, treatments. Data are presented as the mean of three biological replicates, and error bars represent the standard deviation (SD). The significance level is shown as follows: *, *p* < 0.05; *, *p* < 0.01; ***, *p* < 0.001; NS, not significant.

The active ingredients of the tea leaves with or without LP inoculation during anaerobic fermentation are shown in Figure 1. The additive significantly affected the TP, catechin, and total FAA contents (*p* < 0.05). The fermentation days significantly affected the contents of TPs, GA, caffeine, catechins, and total FAA (*p* < 0.05). The interaction between additive treatments and fermentation days significantly influenced catechin and total FAA levels (*p* < 0.05). TP levels in both the CK and the LP treatment decreased before Day 15 and then stabilized. The GA content decreased continuously, but the caffeine content remained stable in both treatments. Compared to the CK, the average total FAA content of the LP treatment decreased by 5.18%, and the average catechin content increased by 4.26%. Notably, by Day 30, the total FAA levels of both treatments attained their maximum values of 8.15% and 7.94%, respectively.

### 3.5 Flavor‐Related FAA

**FIGURE 2:**
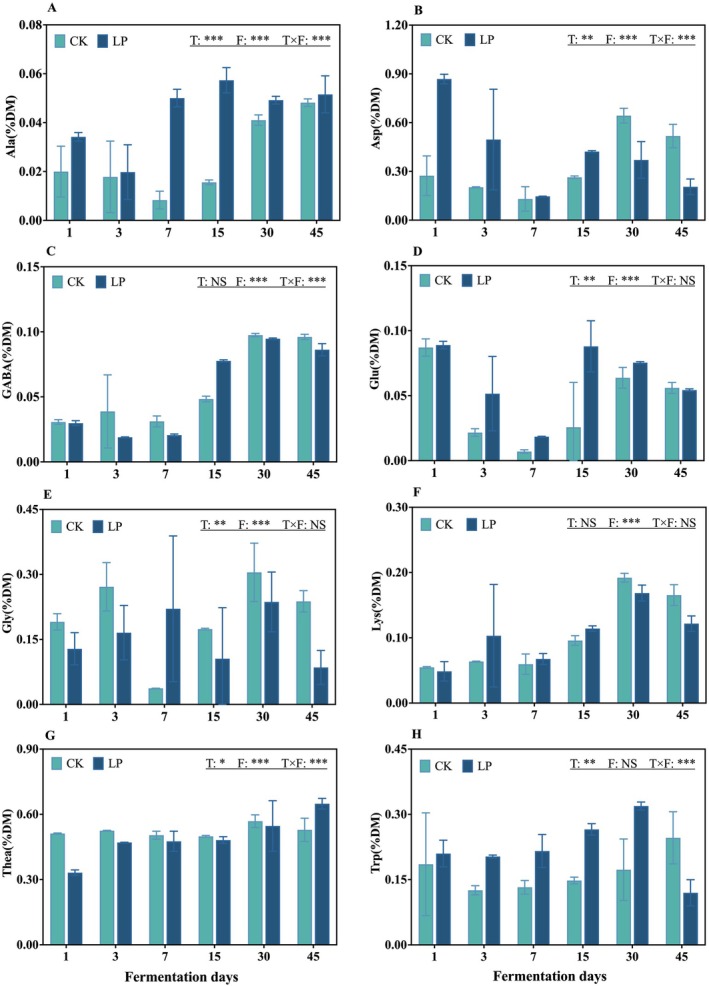
The eight flavor‐related FAA profile of tea leaves with or without *Lactobacillus plantarum* inoculation during anaerobic fermentation. (A) Alanine, Ala; (B) aspartic acid, Asp; (C) γ‐aminobutyric acid, GABA; (D) glutamic acid, Glu; (E) glycine, Gly; (F) lysine, Lys; (G) theanine, Thea; and (H) tryptophan, Trp. CK, control; *F*, fermentation days; LP, *L. plantarum* ; *T* × *F*, interaction between treatment and fermentation days; T, treatments. Data are presented as the mean of three biological replicates, and error bars represent the standard deviation (SD). The significance level is shown as follows: *p < 0.05; **p < 0.01; ***p < 0.001; NS, not significant.

The eight flavor‐related FAA profiles of tea leaves with or without LP inoculation during anaerobic fermentation are shown in Figure 2. The Ala, GABA, and Lys contents first increased and then stabilized (*p* < 0.05). LP significantly increased the Ala content. Asp and Glu levels decreased rapidly on Day 7 in both treatments and then stabilized (*p* < 0.05). There was no significant interaction between treatment and fermentation days in terms of Glu, Gly, and Lys contents (*p* > 0.05). Compared to the CK, LP addition significantly increased the Ala, Glu, Trp, Gly, and Thea contents (*p* < 0.05).

### 3.6 Microbial Community

**FIGURE 3:**
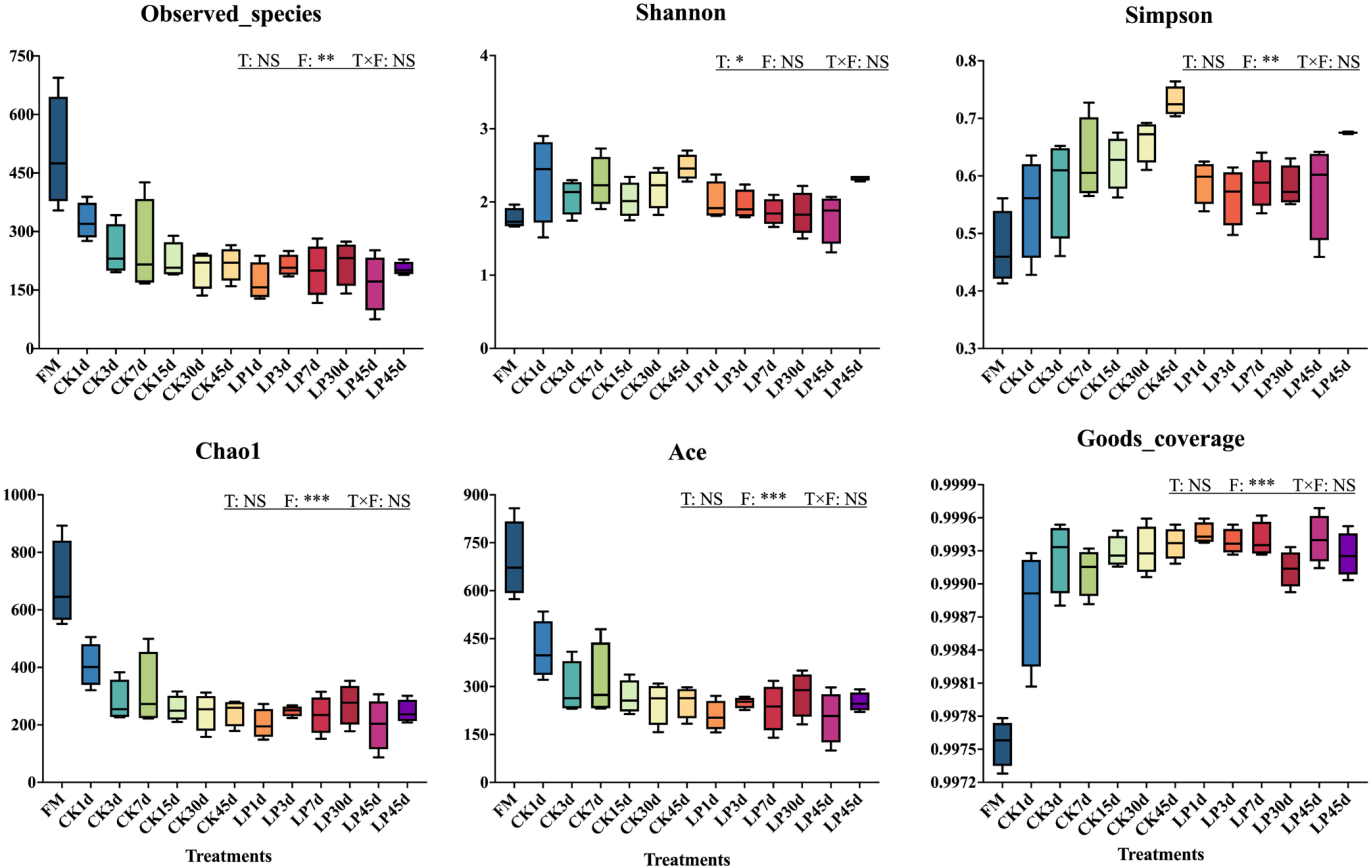
The alpha diversity of bacterial community of tea leaves with or without *Lactobacillus plantarum* inoculation during anaerobic fermentation. CK, control; *F*, fermentation days; FM, fresh matter; LP, *L. plantarum* ; *T* × *F*, interaction between treatment and fermentation days; T, treatments. The significance level is shown as follows: *p < 0.05; **p < 0.01; ***p < 0.001; NS, not significant.

Figure 3 shows the alpha diversity of the bacterial community of tea leaves with or without LP during anaerobic fermentation. LP reduced bacterial abundance and diversity in tea leaves. The coverage index in both treatments was 0.999, and the sequencing depth was very acceptable. Compared to the CK, LP treatment decreased the number of observed species and the Shannon and Simpson indices by Day 45 of fermentation.

**FIGURE 4:**
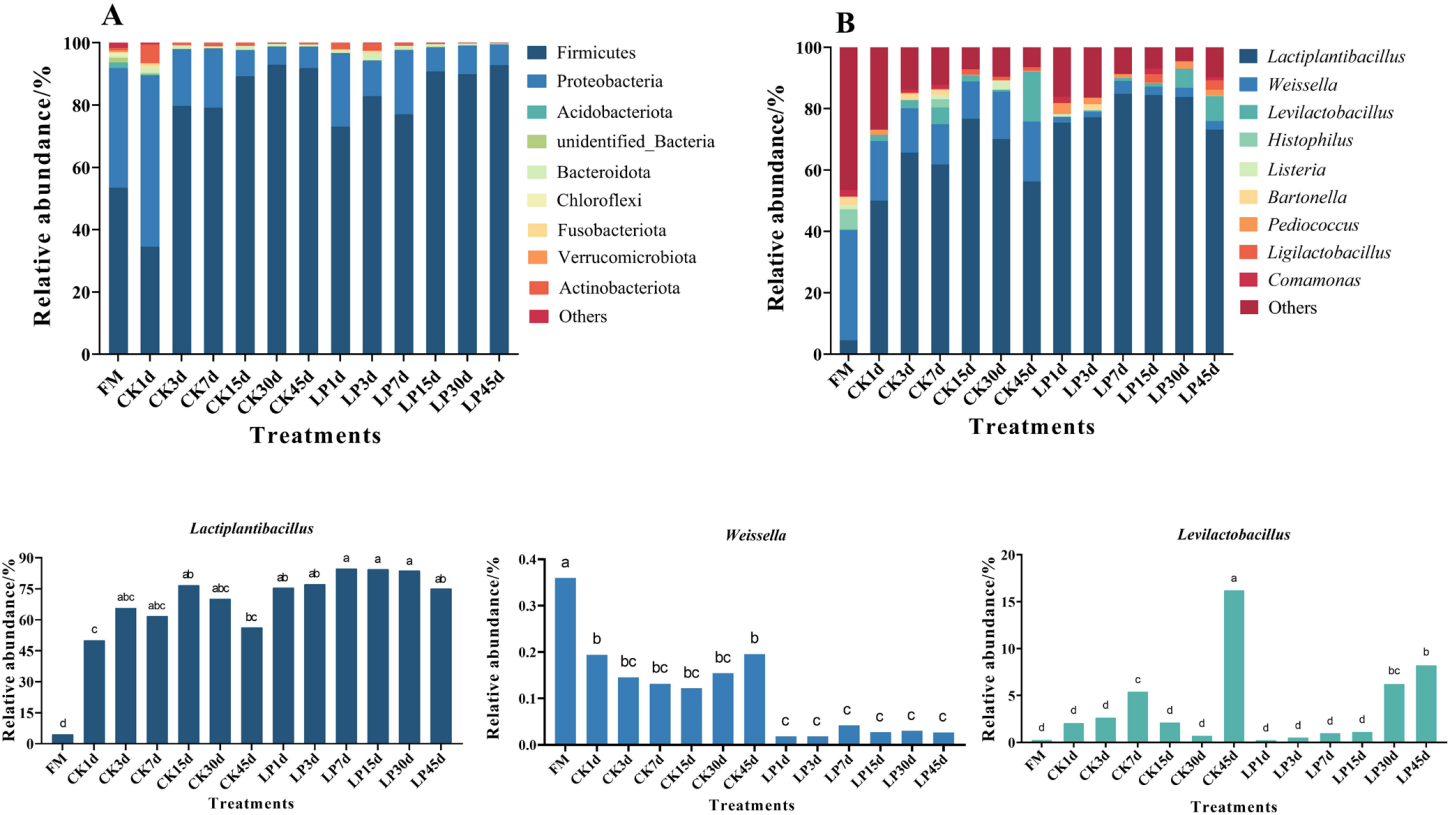
The relative abundance of bacterial communities at the phylum (A) and genus (B) level of tea leaves with *Lactobacillus plantarum* inoculation during anaerobic fermentation. CK, control; FM, fresh matter; LP, *L. plantarum* .

Figure 4 shows the microbial community composition during fermentation. At the phylum level (Figure 4A). Initially, Cyanobacteria, Firmicutes, and Proteobacteria were the dominant phyla in the FM treatment. During the early stage of fermentation, Proteobacteria accounted for a relatively high proportion in 7 days of both treatments, while its relative abundance decreased significantly as fermentation progressed. Conversely, Firmicutes gradually proliferated to become the dominant phylum. By Day 45, the relative abundance of Firmicutes reached 91.84% in the CK treatment and 92.78% in the LP treatment.

At the genus level (Figure 4B), *Weissella* (35.97%) dominated in raw tea leaves, followed by *Histophilus* (6.52%), *Lactiplantibacillus* (4.48%), and *Bartonella* (2.60%). After Day 1 of fermentation, *Lactiplantibacillus* became dominant in both the CK and LP treatments, followed by *Weissella* and *Levilactobacillus*. Dynamic analysis by the fermentation time revealed that the relative *Lactiplantibacillus* abundance first increased and then decreased in both the CK and the LP treatments. Compared to the CK, LP addition significantly increased *Lactiplantibacillus* abundance (*p* < 0.05) to levels 25.39% and 17.01% higher than those of the CK on Days 1 and 45, respectively, and significantly decreased the relative abundance of *Weissella* (*p* < 0.05) to levels 17.57% and 16.83% lower than those of the CK at the same timepoints, respectively.

**FIGURE 5:**
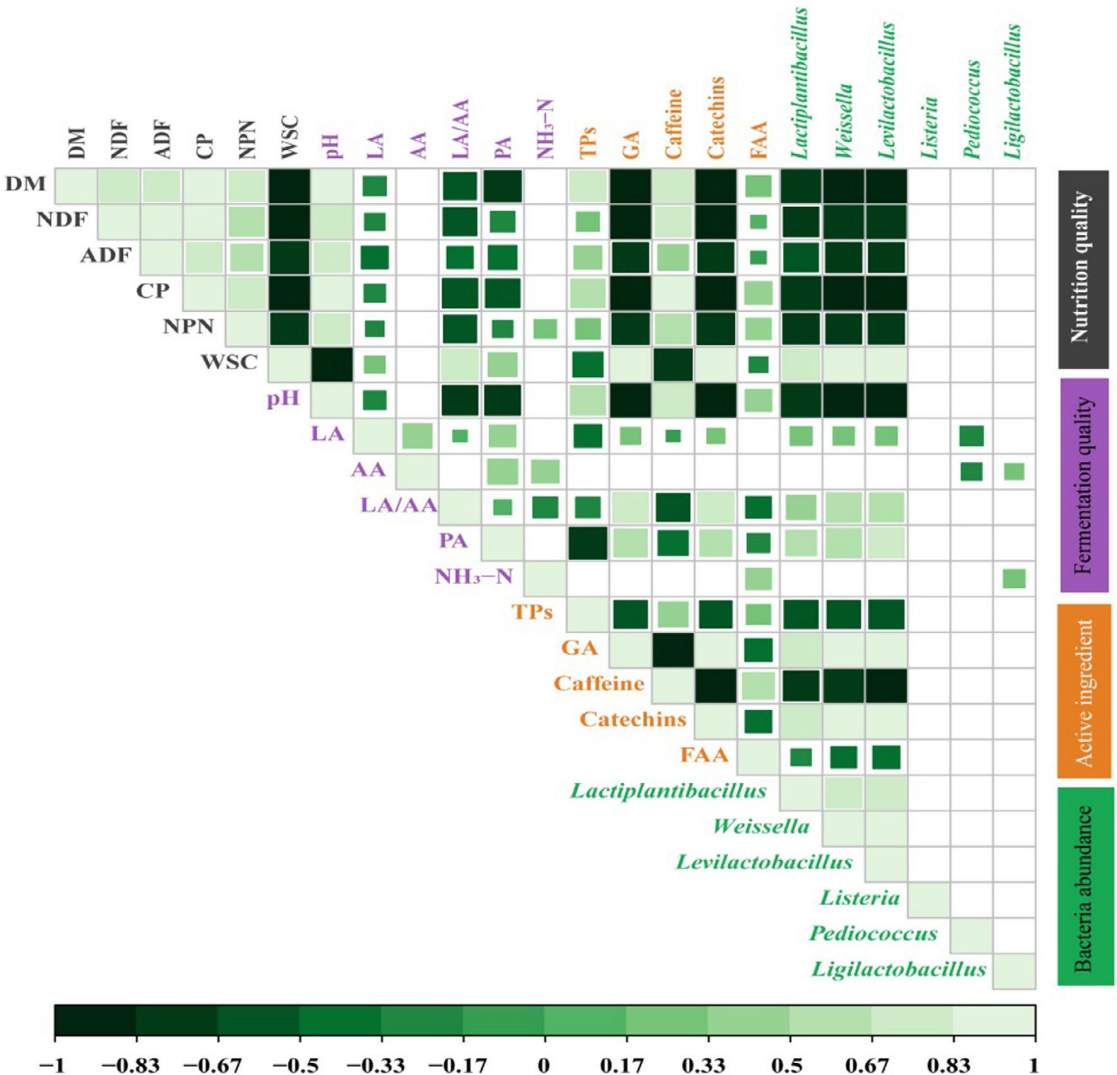
The correlation analysis of microorganisms, active ingredients, fermentation quality, and nutritional quality of tea leaves. Squares with colors indicate significant correlations (*p* < 0.05); the color gradient represents the Pearson correlation coefficient, and the size of the squares represents the absolute value of the correlation coefficient.

The Pearson correlations between six bacterial genera (*Lactiplantibacillus*, *Weissella*, *Levilactobacillus*, *Listeria, Pediococcus*, and *Ligilactobacillus*), the nutritional, and fermentation qualities, and the active ingredients are shown in Figure 5. The catechin and DM levels exhibited significant negative correlations with the relative abundance of *Lactiplantibacillus*, *Weissella*, and *Levilactobacillus* (all *p* < 0.05). Specifically, the NPN and NDF levels were positively correlated with the relative abundance of *Lactiplantibacillus*, but the WSC, TP, and GA contents were negatively correlated.

## 4 Discussion

### 4.1 Nutrient Composition and Fermentation Quality

Generally, LAB preferentially utilize WSC as fermentation substrates to produce organic acids, thereby rapidly decreasing pH, suppressing undesirable microorganisms, and improving nutritional quality and substrate preservation under anaerobic conditions (De Souza et al. 2023). CP content is a critical indicator for evaluating the nutritional value of fermented substrates. During anaerobic fermentation, proteolysis is unavoidable and is primarily mediated by endogenous plant proteases and microbial enzymes, resulting in the degradation of true protein into peptides and amino acids (Feng et al. 2023). In the present study, CP content remained consistently high throughout fermentation in both the CK and LP treatments. This stability may be attributed to the inherently high protein content of tea leaves and the presence of tea tannins, which can form relatively stable tannin‐protein complexes that limit proteolytic activity and reduce protein degradation (Utatsu et al. 2023). Accordingly, the relatively low extent of proteolysis may explain the non‐significant differences in NPN contents between treatments across fermentation stages (Table 3).

Both NDF and ADF contents increased significantly after 45 days of fermentation in CK and LP treatments (Table 3). This increase is most likely attributable to a concentration effect associated with the preferential degradation of non‐fiber components and DM loss during prolonged fermentation. WSC content declined gradually during the initial phase of fermentation and subsequently stabilized after 45 days (Table 3), indicating that WSC was initially consumed as a major fermentation substrate, whereas microbial activity may have been progressively inhibited as fermentation proceeded.

The average LA content did not differ significantly between the LP and CK treatments, whereas the average contents of AA and PA were higher in the CK treatment than in the LP treatment (both *p* < 0.05). Although LP addition increased the relative abundance of LP, its typical LA‐producing capacity was not fully exhibited in the tea leaf matrix. This phenomenon may be associated with an adaptive metabolic shift of this facultatively heterofermentative bacterium under the specific biochemical environment of tea leaves. Bioactive substances in tea, such as TPs, caffeine, and Thea, may inhibit the homofermentative pathway of LP while favoring heterofermentative metabolism. In addition, phenolic acids released during leaf rolling may have contributed to lowering the pH of the fermentation system, further affecting organic acid metabolism (Zhang et al. 2023). Notably, despite the lack of marked LA accumulation, LP treatment effectively suppressed the excess of AA and BA, which may help improve the fermentation quality of summer/autumn tea leaves.

NH_3_–N content reflects the extent of protein degradation during fermentation. Protein hydrolysis releases amino acids, which are subsequently converted to NH_3_–N through deamination. In the present study, NH_3_–N contents in both treatments remained below 10% of total nitrogen throughout the fermentation process (Table 4), which is generally considered indicative of limited protein degradation and is consistent with our previous findings (Lei et al. 2024; Zhou et al. 2024). Notably, the average NH_3_–N content in the LP treatment was significantly higher than that in the CK treatment (*p* < 0.05), and the NH_3_–N contents increased progressively with fermentation time in both treatments (Table 4). Furthermore, a significant positive correlation was observed between total FAA and NH_3_–N (Figure 5), indicating that part of the total FAA was converted into NH_3_–N during fermentation. This finding suggests that prolonged fermentation may promote the deamination of amino acids, leading to NH_3_–N accumulation acceleration. Therefore, appropriately shortening fermentation duration may help reduce the conversion of total FAA into NH_3_‐N during tea leaf fermentation. BA is commonly associated with the activity of undesirable microorganisms, such as *Clostridium*, which can accelerate nutrient decomposition (Zheng et al. 2018). In the present study, BA was not detected in either treatment, indicating that Clostridium activity was effectively suppressed during fermentation (Cheng et al. 2021). This suggests that the observed NH_3_–N accumulation was unlikely to be associated with clostridial spoilage and was more likely derived from amino acid deamination.

### 4.2 Active Ingredients

TPs are the primary antioxidants in tea leaves and are widely regarded as important contributors to the functional value of tea production because of their radical scavenging capacity and multiple bioactivities, including antioxidant, hypoglycemic, antihypertensive, antitumor, and antiarrhythmic effects (Zeng et al. 2024). As shown in Figure 1, TPs contents in both the CK and LP treatments initially increased, which was likely related to the kneading process before fermentation. This process disrupts cellular structures and facilitates the release of active ingredients from tea leaves. However, as fermentation progressed, TP contents gradually decreased in all treatments. This decline might be attributed to the activity of polyphenol oxidase, which catalyzes the oxidation and degradation of catechins and other oxidizable polyphenols into phenolic acids and quinones during the early stages of fermentation (Liu et al. 2022). GA is an important organic acid in tea leaves with strong antioxidant, antibacterial, and anti‐inflammatory properties. GA content in microbially fermented teas is generally relatively high, mainly because the hydrolysis of ester‐type catechins facilitates GA accumulation during fermentation (Shi et al. 2025). However, the GA content decreased significantly in all treatments, which is consistent with the findings of Hou et al. (2023). This reduction may be associated with the limited esterase activity of LAB toward epigallocatechin gallate under anaerobic conditions, resulting in a relatively weak hydrolytic pathway for GA production.

Tea catechins are closely associated with undesirable sensory characteristics, such as bitterness and astringency (Dong et al. 2024). In this study, the catechin contents of the fermented tea leaves decreased significantly from 0.57% to 0.30% during anaerobic fermentation (Figure 1D), in agreement with previous reports (Wang et al. 2023). This reduction likely alleviated the inherent bitterness and astringency of summer/autumn tea and corresponds well with the observed improvement in sensory quality. Previous studies have found that catechin degradation during fermentation occurs through enzymatic transformation into theaflavin‐like compounds and other phenolic derivatives, which lead to a milder and smoother taste while partially retaining the antioxidant capacity of tea (Hu et al. 2022). In contrast, caffeine, a major bioactive alkaloid in tea leaves that contributes substantially to bitterness (Liu et al. 2025), remained stable throughout fermentation (Figure 1C), consistent with the findings of Wen et al. (2023) on pickled tea. This stability is likely due to the structural resistance of the caffeine purine ring to microbial and enzymatic degradation (Luo et al. 2024). As a result, the stability of caffeine helps preserve the flavor intensity and stimulant properties of the final fermented tea product.

### 4.3 Flavor‐Related FAA

Flavor‐related FAA are key contributors to the taste and aroma of tea infusions. According to their sensory characteristics, Flavor‐related FAA can be classified as umami amino acids (Asp, Glu), sweet amino acids (Gly, Thr, Ala), and bitter amino acids (Lys, Arg), and changes in their composition and concentration can substantially affect tea quality (Zhang, Li, et al. 2024; Yu et al. 2025). Generally, a reduction in bitter amino acids helps alleviate bitterness and astringency, whereas an increase in umami and sweet amino acids enhances the freshness, briskness, and mellow sweetness of tea infusions.

Thea, a characteristic amino acid in tea leaves, is an important contributor to umami taste and is also known to counteract some of the excitatory effects of caffeine, such as euphoria and insomnia. In the present research, the Thea content in the LP treatment tended to stabilize during the later stage of fermentation, peaking at 0.65% on Day 45 (Figure 2G), indicating that LP addition may have influenced Thea retention and helped to preserve a core sensory and functional component of tea leaves. In addition, as shown in Figure 2A, Ala average content was significantly higher in the LP treatment than in the CK treatment (*p* < 0.05), which is likely related to the efficient utilization of WSC by LP. This process generates pyruvate as a glycolytic intermediate that serves as a precursor for Ala biosynthesis (Huang et al. 2024). GABA, a non‐protein amino acid with neuroprotective and hypotensive properties (Su et al. 2025), is primarily synthesized through glutamate decarboxylation (Tu et al. 2022). In the present study, the increase in GABA level during anaerobic fermentation was positively correlated with increased Glu content, suggesting that fermentation promoted the directional protein hydrolysis rather than complete degradation. Moreover, the tea fermentation matrix, including polyphenol‐protein interactions and the relatively dense tea leaf cell wall structure, likely constrained extensive protein breakdown, which may increase Glu availability as a substrate for GABA biosynthesis. This could also partly explain the stable CP content in this study. Collectively, these results demonstrate that LP‐mediated anaerobic fermentation effectively regulated flavor‐related FAA profiles, promoted the accumulation of beneficial amino acids, such as Glu, Ala, and GABA, and maintained the Thea stability, which improved both the sensory quality and functional value of fermented summer/autumn tea leaves.

### 4.4 Microbial Community

The alpha diversity analysis (Figure 3) revealed that the Shannon and Simpson indices were consistently lower in the LP treatment than in the CK from Days 3 to 45, indicating that LP inoculation reduced overall bacterial diversity. This reduction reflects the strong competitive ability of LP, which can rapidly utilize nutrients and produce LA, thereby decreasing pH and suppressing the proliferation of undesirable bacteria. This competitive exclusion might stabilize the microbial niche at an early stage of fermentation and help keep the consistency and controllability of fermented tea quality.

As fermentation progressed, Firmicutes became the dominant phylum, while the Proteobacteria decreased significantly (Figure 4A). This community succession was likely driven by the dynamic microenvironment created by LAB fermentation. Residual oxygen present at the early stage might have favored facultative anaerobes within Proteobacteria. However, as oxygen was consumed and organic acids were produced, the fermentation environment gradually became hypoxic and acidic, which was favorable for the Firmicutes proliferation (Du et al. 2025). Importantly, the dominance of Firmicutes, particularly LAB taxa, is closely associated with efficient acidification, reduced protein putrefaction, and suppression of off‐flavor producing microorganisms.

At the genus level (Figure 4B), *Lactiplantibacillus*, *Weissella*, and *Levilactobacillus* were the dominant bacteria. These genera interact through nutrient competition and environmental modification during fermentation. *Weissella* and *Levilactobacillus* are heterofermentative LAB that convert WSC into LA and AA, which can enhance aroma complexity but also lead to excessive sourness when present in high abundances. In contrast, LP is generally regarded as a strong acid‐producing LAB with strong tolerance to low‐pH conditions, and its metabolic behavior may depend on the fermentation matrix. In the LP treatment, the rapid acidic environment established by LP might inhibit the growth of heterofermentative LAB, resulting in lower AA accumulation and a higher LA/AA ratio than that in the CK treatment (Table 4). This microbial interaction could help to explain the milder, more balanced taste observed in LP‐fermented tea, whereas delayed acidification in the CK might allow heterofermentative LAB to persist longer and produce excessive AA, leading to sharper sour notes (Sun et al. 2023). In addition, TPs and GA showed negative correlations with *Lactiplantibacillus* abundance (Figure 5), suggesting that active ingredients released during leaf rolling are likely to modulate LAB dynamics and further shape microbial interactions. Potential pathogenic genera, *Enterobacter* and *Clostridium* were not detected, and *Listeria* abundance was significantly lower in the LP treatment than in the CK. These findings indicate that LP‐mediated acidification effectively suppressed spoilage and pathogenic microorganisms, improving microbiological safety while preventing off‐flavor formation.

## 5 Conclusions

Inoculation with LP shifted the fermentation of summer/autumn tea toward a predominantly homolactic pattern, by an increased LA/AA ratio and reduced abundances of heterofermentative genera. This shift limited AA accumulation and contributed to a more balanced flavor profile. LP fermentation promoted the accumulation of flavor‐related FAA GABA and Ala while maintaining stable levels of Thea and CP. Rapid acidification and competitive exclusion suppressed potential pathogens. In addition, the laboratory‐scale fermentation carried out under constant temperature cannot fully simulate actual processing conditions, the use of a single LP strain makes it difficult to extend the research conclusions to other LAB strains, and the unique phenolic acid compounds in tea itself may also affect the activity of LAB during fermentation. Future studies should validate these effects under pilot‐scale conditions and apply multi‐omics approaches to elucidate tea and microbe interactions.

## Author Contributions

Yimei Luo drafted the manuscript and performed the experiments. Yuangan Qian, Yangwen Ou, and Simin Tian performed the investigation and experiments and data collection. Yixiao Xie, Yulong Zheng, and Jun Hao supervised the study. Hong Sun designed the experiments and revised the manuscript. Fuyu Yang provided project administration and funding acquisition. All the authors have reviewed and approved the final version of the manuscript.

## Funding

This research was funded by the project of Research and Demonstration of the National Key R&D Program of China (2022YFD1300900), Guizhou Provincial Department of Agriculture project of Qian Nong Ji Cai ([2024] 47), Qian Ke He Zhong Da ([2025] 004), Scientific Research Foundation for Talent Introduced in Guizhou University (Gui Da Ling Jun He Zi [2023] 05). 

## Conflicts of Interest

The authors declare no conflicts of interest.

## Data Availability

The data that support the findings of this study are available on request from the corresponding author. The data are not publicly available due to privacy or ethical restrictions.
